# Pneumococcal infective endocarditis in Brazil: a multicenter study on a severe condition

**DOI:** 10.1016/j.bjid.2024.103837

**Published:** 2024-07-04

**Authors:** Roxana Flores Mamani, Rinaldo Focaccia Siciliano, Claudio Querido Fortes, Paulo Vieria Damasco, Cristiane da Cruz Lamas

**Affiliations:** a Fundação Oswaldo Cruz, Instituto Nacional de Infectologia Evandro Chagas, Rio de Janeiro, RJ, Brazil; b Universidade de São Paulo, Faculdade de Medicina, Hospital das Clínicas (HCFMUSP), Instituto do Coração (InCor), São Paulo, SP, Brazil; c Universidade Federal do Rio de Janeiro (UFRJ), Rio de Janeiro, RJ, Brazil; d Universidade do Estado do Rio de Janeiro (UERJ), Rio de Janeiro, RJ, Brazil; e Universidade Federal do Estado do Rio de Janeiro (Unirio), Rio de Janeiro, RJ, Brazil; f Instituto Nacional de Cardiologia, Rio de Janeiro, RJ, Brazil

**Keywords:** *Streptococcus pneumoniae*, Infective endocarditis, Surgery, Mortality

## Abstract

**Background:**

Streptococcus pneumoniae bacteremia may result in Infective Endocarditis (IE). In the pre-antibiotic era, it caused 10 %‒15 % of IE, decreasing to < 3 % after penicillin availability. Although infrequent, it causes aggressive disease.

**Methods:**

Retrospective analysis of endocarditis databases, prospectively implemented in 4 Brazilian institutions, 2005‒2023.

**Results:**

From the prospective cohorts comprising 2321 adult patients with IE, we identified 11 (0.47%) with pneumococcal IE. Males represented 7/11 and mean age was 54 years (22‒77). All had native valve involvement; perivalvular abscess was present in 6/11. Only one patient had concurrent meningitis. Beta-lactams were the antibiotics used in 10/11. All had surgical indication, but only 6 had it, as the others were seriously ill. Overall, in hospital mortality was 6/11, but only 1/6 of those who underwent surgery died, compared to 5/5 of those who had an indication for surgery and did not have it.

**Conclusions:**

The high mortality rates and need for surgical intervention emphasize the need to promptly identify and manage pneumococcal endocarditis. Physicians ought to recommend vaccination to all patients at risk for severe pneumococcal disease.

*Streptococcus pneumoniae* is a Gram-positive encapsulated coccus which belongs to the normal microbiota of the upper respiratory tract.^1^^,^^2^ It is the leading etiologic agent of community-acquired pneumonia, meningitis, sinusitis, and otitis media. When it causes bacteremia, secondary complications such as endocarditis, arthritis, or meningitis may occur.^3^^,^^4^

Invasive Pneumococcal Disease (IPD) is an infection confirmed by isolating *S. pneumoniae* from sterile sites. It remains a frequent condition in adults in some predisposed groups, especially elderly patients, those with diabetes, cirrhosis, chronic renal failure, splenectomy, or functional asplenia, and HIV positive patients.^3^, ^4^, ^5^, ^6^ Despite this, infective endocarditis rarely results from pneumococcal bacteremia. Marrie et al., in Canada, evaluated 3251 adult patients with IPD and verified that only 28 (0.3 %) developed endocarditis; ^7^ these patients were more likely to use illicit drugs and have a higher severity of illness at presentation (higher rate of altered mental status and need for intensive care), and higher mortality (39.3% vs. 14.7 % respectively). ^7^ However, no other major risk factors were identified for endocarditis in adults with IPD.^7^ In a prospective, international, observational study, eight of 844 patients hospitalized with *S. pneumoniae* bacteraemia only 5 developed IE (0.6 %).^8^ They did not show greater mortality (1/5 or 20 % with IE died) or a different clinical pattern, and the bacterial isolates from the endocarditis cases showed no specific virulence traits or adherence capabilities.^8^ In a study on IPD in Israel,^9^ from 2009‒2019, 23/4119 (0.6 %) were endovascular pneumococcal infections; inhospital mortality was 21.7 %.^9^ In Australia, a retrospective study on pneumococcal bacteremia in adults^10^ included from 2011–2020 found that only 2/300 (0.7 %) were endocarditis cases.^10^ In Brazil, a recent study on IPD in HIV positive patients in a infectious diseases referral center in Brazil, from 2005‒2020, showed that none of the 55 patients had IE.^5^

From a historical perspective, *S. pneumoniae* used to frequently cause acute endocarditis in the pre antibiotic era,^11^ but the main causative germ shifted to *S.aureus* post the availability of penicillins in medical practice; moreover, since penicillin became available, the frequency of pneumococcal endocarditis decreased from 10 %–15 % to < 3 %.^12^ Indeed, contemporary studies from laboratories which have dealt with streptococcal bacteremia and correlated these infections to endocarditis diagnosis, have reported different weights to the diverse streptococcal species and have found *S. pneumoniae* as non-associated to IE.^13^^,^^14^ This has led to *S.pneumoniae* being excluded from the typical viridans group as major criterion in the Duke-ISCVID criteria update in 2023. However, although pneumococcal IE is relatively uncommon, it is a severe disease, with inhospital mortality rates that vary from 20.7 % to 39.3 %.^7^^,^^9^^,^^15^^,^^16^ Furthermore, it may occur in all age groups, and may be associated with another severe infection, meningitis.^17^^,^^18^

No Brazilian series of pneumococcal IE has been published so far. We have, therefore, set out to study pneumococcal endocarditis at 4 sites in Brazil, to estimate its relative frequency, characteristics and outcomes.

This was a retrospective analysis of endocarditis databases, prospectively implemented, with a *post hoc* study driven by analysis of cases of pneumococcal endocarditis. Consecutive and prospective adult patients with definite endocarditis according to the modified Duke criteria were studied from 2005 to 2023 in 4 Brazilian institutions, all public hospitals, two of which were cardiac referral centers, and two university hospitals. Centers participating in this study were Instituto Nacional de Cardiologia (INC), Hospital Universitário Clementino Fraga Filho, from the Federal University of Rio de Janeiro (UFRJ), Hospital Universitário Pedro Ernesto (HUPE/UERJ), all located in Rio de Janeiro city, and Instituto do Coração (InCor), part of the University Hospital of São Paulo, located in the city of São Paulo. This is, therefore, a convenience sample. The study was approved by each local Ethics Committee.

Clinical and laboratory data were collected from patients’ notes. Microbiological data were collected from the results provided by the Bacteriology Laboratories in an automated system and from internal laboratory records of each institution. Isolation of *Streptococcus pneumoniae* by direct inoculation of clinical samples (blood) onto enriched culture media (chocolate agar and blood agar) and incubation at 5 % CO_2_ and 35±1 °C temperature, within 72 hours of receiving the samples, previously inoculated into automated culture flasks (Bactec/Becton Dickson) for up to 5 days. Antimicrobial susceptibility testing was carried out using the agar diffusion method (disc diffusion), and determination of the minimum inhibitory concentration by agar diffusion with a gradient strip. The interpretative criteria of the CLSI ‒ Clinical and Laboratory Standards Institute were followed. Molecular tests and serotyping were not routinely done.

Study variables: The study variables were obtained from each cohort's database and supplemented with data extracted from the electronic medical records. The following variables were collected: sex at birth, age at diagnosis, valve affected, presence of perivalvular abscess, vegetation size, embolization to the central nervous system or spleen, antibiotics used, duration of treatment, whether surgery was indicated, creatinine levels on admission, days of hospitalization and in hospital death outcome. The only variable obtained retrospectively was the Euroscore on admission.

Descriptive statistical analysis (frequencies) and mean, standard deviation and amplitude statistics were performed using the Microsoft Excel software.

From the prospective cohorts comprising 2321 adult patients with infective endocarditis, we identified eleven with Pneumococcal Endocarditis (PE) from 2005 to 2023, from the afore mentioned Brazilian institutions. In institution A, the frequency of PE in adults was of 7/1154 (0.6 %) of cases, in institution B, of 2/502 (0.4 %), in institution C 1/539 (0.2 %) and in institution D, 1/126 (0.7 %); mean frequency was therefore of 11/2321 (0.5 %). Clinical and laboratory aspects of the cases are shown on Table 1.

**Table 1:** Cases of pneumococcal endocarditis in four Brazilian medical centers, 2005‒2023.

Cases	Year/ Site	Gender	Age (years)	Affected valves	Perivalvular abscess	Largest vegetation size (in mm)	Antibiotics used	Total duration of treatment (days)	Was surgery indicated?	Was surgery done?	Serum Creatinine (mg/dL)	Euro SCORE II	Length of hospitalization (days)	Inhospital death?
**1**	2005 A	Female	51	Native AV	**No**	**NA**	Vancomycin + gentamicin + ceftriaxone	30	Yes	Yes	0.7	0.86 %	30	No
2	2007 C	Male	22	Native MV	Yes	20 mm	Penicillin + gentamicin + ampicillin	79	Yes	Yes	0.8	1.7 %	52	No
3	2007 B	Male	51	Native AV and MV	Yes	25 mm	Penicillin + gentamicin	41	Yes	Yes	0.8	3.21 %	46	No
4	2008 A	Male	75	Native AV	No	13 mm	Penicillin + gentamicin	11	Yes	No^a^	2.1	38.61 %	11	Yes
5	2008 A	Female	55	Native TV	No	7mm	Vancomycin	27	Yes	No^a^	2.2	31.31 %	27	Yes
6	2011 A	Female	77	Native AV and MV	No	NA	Ceftriaxone + gentamicin	12	Yes	Yes	1.8	21.18 %	12	Yes
7	2011 B	Male	31	Native AV	Yes	NI	Ampicilin + gentamicin	42	Yes	Yes	1.1	1.24 %	55	No
8	2016 A	Male	39	Native AV and MV	No	4 mm	Ceftriaxone + clarithromycin + oxacillin	37	Yes	Yes	0.7	8.2 %	37	No
9	2016 A	Male	65	Native AV	Yes	14 mm	Ceftriaxone	NI	Yes	No^a^	1.35	10.78 %	17	Yes
10	2017 D	Female	64	Native MV	Yes	NI	Vancomycin + ceftriaxone	6	Yes	No^a^	1.48	24.72 %	6	Yes
11	2022 A	Male	71	Native AV and MV	Yes	> 10 mm	Ceftriaxone	4	Yes	No^a^	3.17	18.67 %	4	Yes

Institutions A, B, C and D to be disclosed after review.^b^

NA, Not Available; mm, Milimetres; AV, Aortic Valve; MV, Mitral Valve; TV, Tricuspid Valve.

a Seriously ill or critically ill.

b Institutions A,B,C and D are A=INCOR=Instituto do Coração, Universidade de São Paulo; B= INC=Instituto Nacional de Cardiologia, Rio de JaneiroC=UFRJ=Universidade Federal do Rio de Janeiro; D= HUPE/UERJ=Hospital Universitario Pedro Ernesto, Universidade do Estado do Rio de Janeiro

The majority, 7/11 were male, with a mean age of 54 years (range 22‒77). Only one patient was splenectomized. All had native valve left-sided native valve IE, except for one, who had native tricuspid valve involvement. None had previous valvular disease. Perivalvular abscess was present in 6/11. The size of the vegetation was measured in 7 out of 11 patients (for 2 patients the information was not available, and 2 did not present vegetations) with an average of 13 mm (4‒25). Only one of the patients presented embolization to the central nervous system, and none presented emboli to the spleen, or vertebrae.

Beta lactams were the antibiotics used in 10/11 patients, and 1 patient used vancomycin; gentamicin was associated in 6 patients. Mean duration of antibiotic therapy was 29 days (4‒79) in 10/11 (in one patient duration of therapy was not available, and two patients died quickly, at 4 and 6 days of hospitalization). All patients had surgery indicated, but only six patients effectively underwent surgery, as 5/11 were not clinically well enough to be operated. Euroscore II, as a percent chance of post operative death, was calculated for all patients and presented in Table 1. Average length of stay was 27 days (4‒55), and 6 out of 11 died; only one patient of the 6 who were operated died, vs. 5 of the 5 of those who had an indication for surgery and did not have it.

Our multicenter study is the first to describe pneumococcal endocarditis in Brazil. Similarly to what has been reported in contemporary series, from Canada,^7^ Israel,^9^ Australia^10^ and European countries,^9^^,^^15^, ^16^, ^17^, ^18^ we found that pneumococcal IE was infrequent . However, it was associated with a severe status, destructive disease, and death in over half our patients (6/11); all five patients who had indication to operate, but were too poorly to have surgery, died. Daudin et al.^19^ found patients with pneumococcal IE, compared to those with other causes of IE, had more heart failure (64.3% vs. 23.2 %; *p* < 0.01), shock (53.6% vs. 23.2 %; *p* < 0.01), need for cardiac surgery (64.3 %) but an inhospital mortality rate of 7.1 %, with surgery having a protective effect.^19^ We emphasize that all but one of our patients who had surgery died.

Only 4 of our 11 patients were elderly and only one presented comorbidities which involved immunosupression (splenectomy); none had diabetes, HIV, cancer, or use of immunossupressive drugs. As a matter of fact, a case-control study, comparing 28 patients with pneumococcal IE with those with other causes of IE (paired only by order of hospital admission) in a teaching center in France,^19^ found some differences between groups: in pneumococcal IE patients, there was a higher proportion of alcoholism (39.3% vs. 10.7 %; *p* < 0.01) and smoking (60.7% vs. 21.4 %; *p* < 0.01). Another older French study found that half of the 30 patients with IE suffered from chronic alcoholism.^15^ A study on 111 cases of pneumococcal IE^16^ comprising 24 patients from the Spanish cohort (2004 to 2013) and 87 cases from literature review (2000‒2013), showed liver disease was present in 27.9 %, immunosuppression in 10.8 % and splenectomy or asplenia in 8.1 %.^16^ We have no information on alcohol and smoking in our patients, but 4 of 11 were young and previously healthy. Only one of our patients (Case 9) had had previous splenectomy (as a child), and none of the others had liver disease or immunosupression.

All our patients had pneumococcal IE on native valves but abscesses were frequent, occuring in 6/11; in Egea et al.,^16^ abscesses were found in only 8.1 % of the 111 patients, and in 7/30 (23.3 %) patients in Lefort et al.^15^ This may be due to a referral bias, as two of our centers were reference for cardiac surgery.

Only one of the patients in our series had meningitis (Case 9, who actually had the Austrian syndrome), differently from others.^15^, ^16^, ^17^, ^18^ This may be because the association is rare (< 5 %) and we have only 11 patients in our series. An older series, with 30 adult patients reviewed retrospectively between 1991 and 1998 in France, found that 12 (40 %) had associated meningitis.^15^ Meningitis concomitant with endocarditis was described as a risk factor associated with death on multivariate analysis with an OR, of 4.3; in this study, meningitis was present in 40.5 % of the 111 patients,^16^ and Austrian syndrome in 26.1 % of them.^16^ Other recent series have dealt with this association. A prospective nationwide observational cohort study on patients with community-acquired bacterial meningitis in the Netherlands from 2006 to 2012 identified endocarditis in 24 of 1025 episodes (2 %); *S.pneumoniae* accounted for 13 of those (54.2 %).^17^ Another study looked at the association of IE and meningitis: it was conducted in France, where 2 databases, one from 2008 with patients with IE in seven French regions, and the other with community-acquired acute bacterial meningitis, from 69 French hospitals in 2013–2014, were matched.^18^ Among the 1030 patients from the merged cohorts, there were 42 (4.1 %) patients who had both IE and meningitis; of these, 18 (42.9 %) had *S.pneumoniae* as the causative microorganism and 7 (16.7 %) patients with IE and bacterial meningitis presented an Austrian syndrome.^18^ All patients with *S.pneumoniae* IE and meningitis were at risk for invasive pneumococcal disease (alcoholism, smoking, diabetes mellitus and AIDS in varying proportions), and had a severe presentation (septic shock, heart failure, or coma), but, surprisingly, none died.^18^

Although pneumococcal vaccination is crucial in elderly patients and those with comorbidities, in our small series, 7 of our cases had no formal indication for it and for the other four, vaccination status was not recorded. Only one of our patients had the pneumococcal serogroup determined (it was the 22F).^20^ We found only one study on pneumococcal serogroups in endovascular infections (including IE) involving a fair amount of patients; in this Israeli study, 18/23 had their vaccine status known but only 4 (22.2 %) received the vaccine prior to their pneumococcal endovascular infection.^9^ All medical specialties must reinforce the need to vaccinate susceptible patients, as pneumococcal endocarditis is rare, but pneumococcal pneumonia is a very common condition, of potential severity.^3^, ^4^, ^5^, ^6^, ^7^, ^8^, ^9^, ^10^^,^^15^^,^^16^

Limitations of our study are that results may not be generalizable, as the endocarditis cohorts from which patients with pneumococcal endocarditis were described are followed up in referral centers, either surgical or university hospitals. Besides, the sample is a convenience sample and the number of pneumococcal endocarditis is very small, making strong assumptions unfeasible.

In conclusion, although pneumococcal IE is rare, it should be sought for in patients with severe pneumococcal infections, especially if heart murmur and emboli are identified. The relativelly high mortality and need for surgical intervention emphasize the need to promptly identify and manage pneumococcal endocarditis. Lastly, physicians ought to recommend vaccination to all patients at risk for severe pneumococcal disease.

## Conflicts of interest

The authors declare no conflicts of interest.

## References

[1] Arguedas A., Trzciński K., O'Brien K.L., Ferreira D.M., Wyllie A.L., Weinberger D. (2020). Upper respiratory tract colonization with Streptococcus pneumoniae in adults. Expert Rev Vaccines.

[2] Ganaie F., Saad J.S., McGee L., van Tonder A.J., Bentley S.D., Lo S.W. (2020). A new pneumococcal capsule type, 10D, is the 100th serotype and has a large cps fragment from an oral Streptococcus. MBio.

[3] Anderson R., Feldman C. (2023). The global burden of community-acquired pneumonia in adults, encompassing invasive pneumococcal disease and the prevalence of its associated cardiovascular events, with a focus on pneumolysin and macrolide antibiotics in pathogenesis and therapy. Int J Mol Sci.

[4] Weight C.M., Jochems S.P., Adler H., Ferreira D.M., Brown J.S., Heyderman RS. (2021). Insights into the effects of mucosal epithelial and innate immune dysfunction in older people on host interactions with Streptococcus pneumoniae. Front Cell Infect Microbiol.

[5] Mamani R.F., López T.A., Jalo W.M., Alves M.R., Nunes E.P., Pereira M.S. (2023). Invasive pneumococcal disease in people living with HIV: a retrospective case-control study in Brazil. Trop Med Infect Dis.

[6] Sangil A., Xercavins M., Carballeira M.R., Andrés M., Riera M., Espejo E. (2015). Impact of vaccination on invasive pneumococcal disease in adults with focus on the immunosuppressed. J Infect.

[7] Marrie T.J., Tyrrell G.J., Majumdar S.R., Eurich D.T. (2018). Risk factors for pneumococcal endocarditis. Eur J Clin Microbiol Infect Dis.

[8] Kan B., Ries J., Normark B.H., Chang F.Y., Feldman C., Ko W.C., Rello J., Snydman D.R., Yu V.L., Ortqvist A. (2006). Endocarditis and pericarditis complicating pneumococcal bacteraemia, with special reference to the adhesive abilities of pneumococci: results from a prospective study. Clin Microbiol Infect.

[9] Kenig A., Oster Y., Cohen-Poradosu R., Reisenberg K., Wieder-Finesod A., Hershman-Sarafov M., Oren I., Weber G., Dagan R., Regev-Yochay G., Strahilevitz J (2022). Characteristics of endovascular pneumococcal infections; a decade of nationwide surveillance study. Eur J Clin Microbiol Infect Dis.

[10] Colaco C.M.G., O'Sullivan M., Zhang H., Huynh D., Sintchenko V., Oftadeh S., Gilbert G.L., Dotel R. (2023). Pneumococcal bacteraemia in adults over a 10-year period (2011-2020): a clinical and serotype analysis. Intern Med J.

[11] Locke E.A (1924). Pneumococcus endocarditis. Trans Am Climatol Clin Assoc.

[12] Aronin S.I., Mukherjee S.K., West J.C., Cooney E.L. (1998). Review of pneumococcal endocarditis in adults in the penicillin era. Clin Infect Dis.

[13] Chamat-Hedemand S., Dahl A., Østergaard L., Arpi M., Fosbøl E., Boel J. (2020). Prevalence of infective endocarditis in streptococcal bloodstream infections is dependent on streptococcal species. Circulation.

[14] Seo H., Hyun J., Kim H., Park S., Chung H., Bae S. (2023). Risk and outcome of infective endocarditis in Streptococcal bloodstream infections according to Streptococcal species. Microbiol Spectr.

[15] Lefort A., Mainardi J.L., Selton-Suty C., Casassus P., Guillevin L., Lortholary O. (2000). Streptococcus pneumoniae endocarditis in adults. A multicenter study in France in the era of penicillin resistance (1991–98). The Pneumococcal Endocarditis Study Group. Medicine.

[16] de Egea V., Muñoz P., Valerio M., de Alarcón A., Lepe J.A., Miró J.M. (2015). and the GAMES Study Group. Characteristics and outcome of streptococcus pneumoniae endocarditis in the XXI century: a systematic review of 111 cases (2000-2013). Medicine.

[17] Lucas M.J., Brouwer M.C., van der Ende A., van de Beek D. (2013). Endocarditis in adults with bacterial meningitis. Circulation.

[18] Béraud G., Tubiana S., Erpelding M.L., Le Moing V., Chirouze C., Gorenne I. (2022). AEPEI study group; COMBAT study group. Combined bacterial meningitis and infective endocarditis: when should we search for the other when either one is diagnosed?. Infect Dis Ther.

[19] Daudin M., Tattevin P., Lelong B., Flecher E., Lavoué S., Piau C. (2016). Characteristics and prognosis of pneumococcal endocarditis: a case-control study. Clin Microbiol Infect.

[20] Cabral-Oliveira G.G., Motta I.C.M., Pereira-Ribeiro M., Damasco P.V., Guaraldi L.M. (2023). Invasive disease by Streptococcus pneumoniae: a case report and a discussion about the immunization rates in older adults in Brazil. BJHBS.

